# Coincidence of acral peeling skin syndrome and Nagashima‐type palmoplantar keratosis in a Japanese pedigree with acral skin peeling

**DOI:** 10.1111/1346-8138.17422

**Published:** 2024-08-12

**Authors:** Toshihide Higashino, Mayu Konomi, Akiharu Kubo, Hiroshi Horinosono, Yoshinori Miura

**Affiliations:** ^1^ Graduate School of Business Administration Keio University Kanagawa Japan; ^2^ Department of Dermatology Self‐Defense Forces Central Hospital Tokyo Japan; ^3^ Department of Human Genetics, Graduate School of Medicine The University of Tokyo Tokyo Japan; ^4^ Department of Psychiatry Self‐Defense Forces Central Hospital Tokyo Japan; ^5^ Division of Dermatology, Graduate School of Medicine Kobe University Hyogo Japan

**Keywords:** corneodesmosome, East Asian, genodermatosis, *SERPINB7*, *TGM5*

## Abstract

Acral peeling skin syndrome (APSS; MIM 609796) is a rare genodermatosis characterized by painless focal cutaneous exfoliation of the dorsal hands and feet, typically displaying autosomal recessive inheritance. While cases associated with a founder mutation in *TGM5* are relatively common in European Caucasian populations, no APSS cases have been reported from Japan or other East Asian countries. In contrast, Nagashima‐type palmoplantar keratosis (NPPK; MIM 615598), caused by variants in *SERPINB7*, is relatively common in East Asia due to founder mutations. We describe a 27‐year‐old Japanese woman with spontaneous focal cutaneous exfoliation of the dorsal hand following prolonged glove use, indicative of APSS. Histopathological examination revealed a cleft between the stratum corneum and stratum granulosum and within the horny layer of the epidermis, supporting this diagnosis. However, her mother and maternal uncle exhibited similar symptoms, and there was no reported consanguinity in the patient's parents or grandparents, prompting suspicion of an autosomal dominant genodermatosis. Whole‐genome sequencing (WGS) revealed compound heterozygous variants in *TGM5* (c.1037G>A and c.684 + 1G>A) as suspected causative variants in the patient, leading to an APSS diagnosis, the first reported in East Asia. On the other hand, her mother and maternal uncle were diagnosed with NPPK due to compound heterozygous pathogenic variants in *SERPINB7* (c.796C>T and c.455‐1G>A). This case highlights the complexity of diagnosing skin disorders when multiple genodermatoses with similar phenotypes exist within a pedigree. Comprehensive genetic analyses, such as whole‐exome sequencing and WGS, are invaluable for identifying causative variants in such complex cases.

## 1 INTRODUCTION

Peeling skin syndrome (PSS [MIM 270300]) is a rare genodermatosis characterized by asymptomatic superficial exfoliation due to the separation of the stratum corneum in the epidermis.^1^ PSS is a highly heterogeneous syndrome and generally classified into localized and generalized forms.^1^ Acral PSS (APSS [MIM 609796]) is a variant of localized PSS with peeling limited to the palms, soles, and dorsal surfaces of the hands and feet, occasionally involving the limbs.^2^ Thus far, there has been no report of an APSS case in any East Asian countries. Conversely, Nagashima‐type palmoplantar keratosis (NPPK; MIM 615598) is a relatively common autosomal recessive genodermatosis^3^, ^4^ in East Asia and may also cause peeling of palmoplantar skin.^5^ We report the first Japanese case of APSS with maternal family members having NPPK.

## 2 CASE REPORT

**FIGURE 1:**
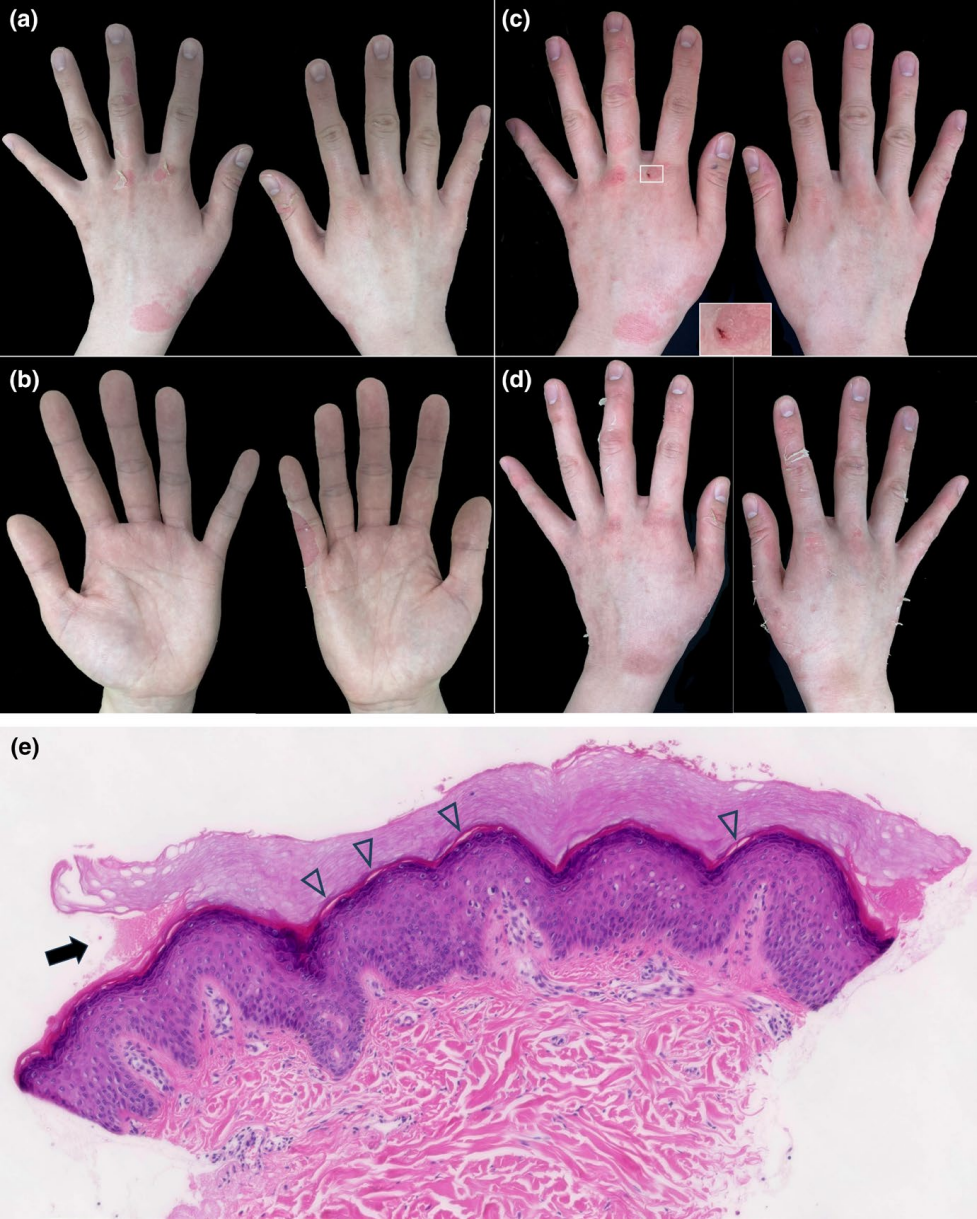
Clinical and histological presentations of the patient. (a, b) Spontaneous focal cutaneous exfoliation of the dorsal hands occurred after wearing surgical gloves for 2 h. (c) After 3 days, abrasions were occasionally observed on the peeled regions. One of the abrasions, outlined by a white square, is magnified. (d) Two weeks after the first visit, the initial skin peeling had healed without scarring, but the skin peeling recurred after another instance of wearing surgical gloves for 2 h. (e) Histopathology showing a cleft between the stratum granulosum and corneum (arrow) and within the horny layer (arrowheads) of the epidermis (hematoxylin and eosin, ×400).

The patient, a 27‐year‐old Japanese woman, provided consent to publish her clinical information, including images. She presented with spontaneous focal cutaneous exfoliation of the dorsal hands after wearing surgical gloves for 2 hours. The peeling was painless, revealing red underlying skin, with no exfoliation on the palms except for the extension of dorsal skin peeling (Figure 1a,b). Her nails, hair, teeth, and other skin and mucous membranes were normal. Occasionally, abrasions were observed in the peeled regions 3 days later (Figure 1c). Two weeks after her first visit, the initial symptoms resolved without scarring, but the skin peeling reoccurred after wearing surgical gloves again (Figure 1d). Since age 18, she has experienced skin peeling after wearing leather or surgical gloves for a few hours. Additionally, she has had focal cutaneous exfoliation of the dorsal ankle after long‐distance walking since childhood. She had no other medical history or complications.

Histopathological examination of the dorsal hand showed a cleft between the stratum corneum and stratum granulosum, and within the horny layer of the epidermis (Figure 1e), with no inflammatory changes or significant abnormalities in the dermis and epidermis. Based on these findings, APSS was suspected.

**FIGURE 2:**
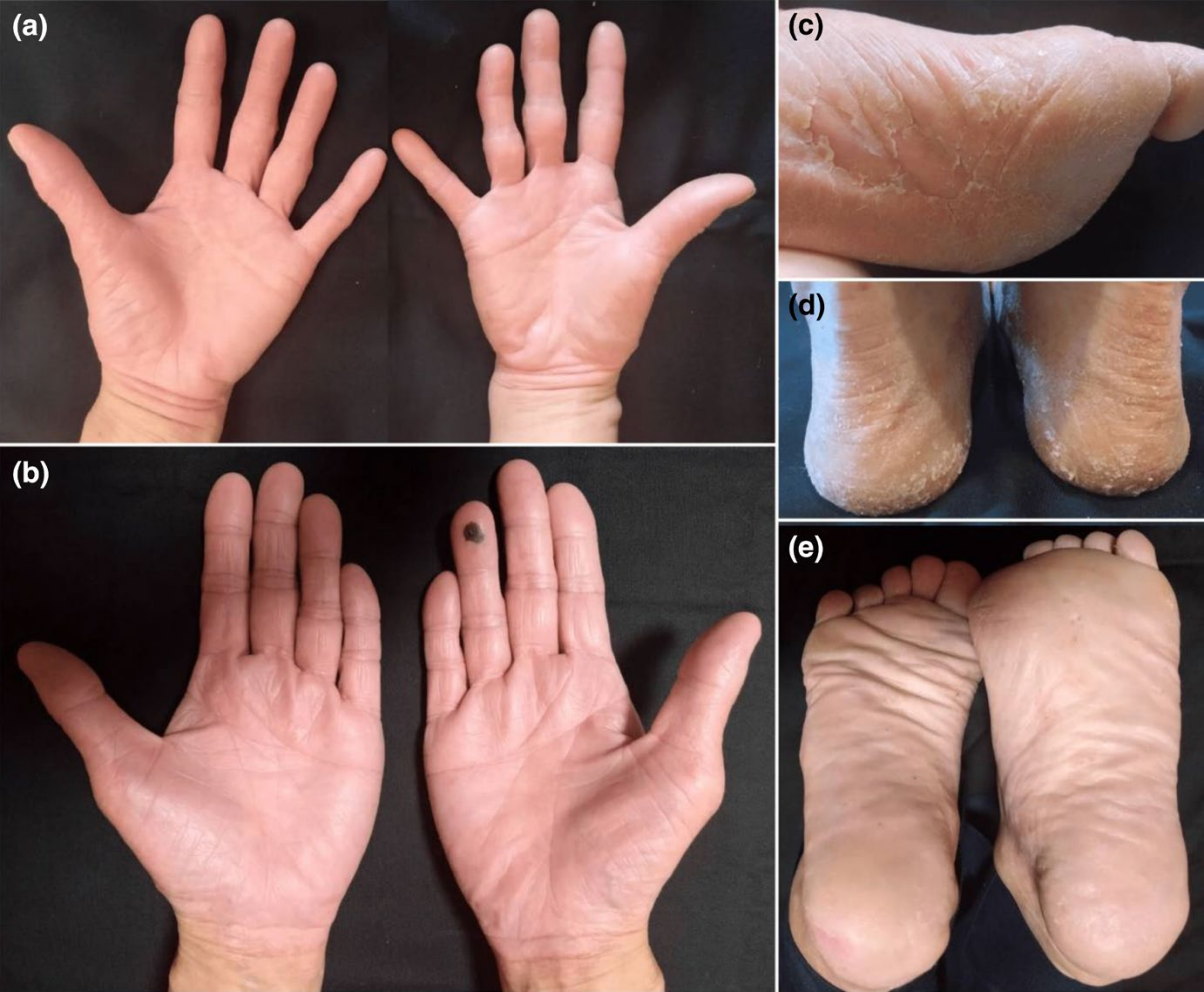
Clinical presentations of the patient's maternal family members. (a, b) Both the mother and maternal uncle show slightly reddish and hyperkeratotic palms. (c, d) The mother exhibited large thick scales on her soles. (e) The maternal uncle also showed hyperkeratosis but with few scales.

**FIGURE 3:**
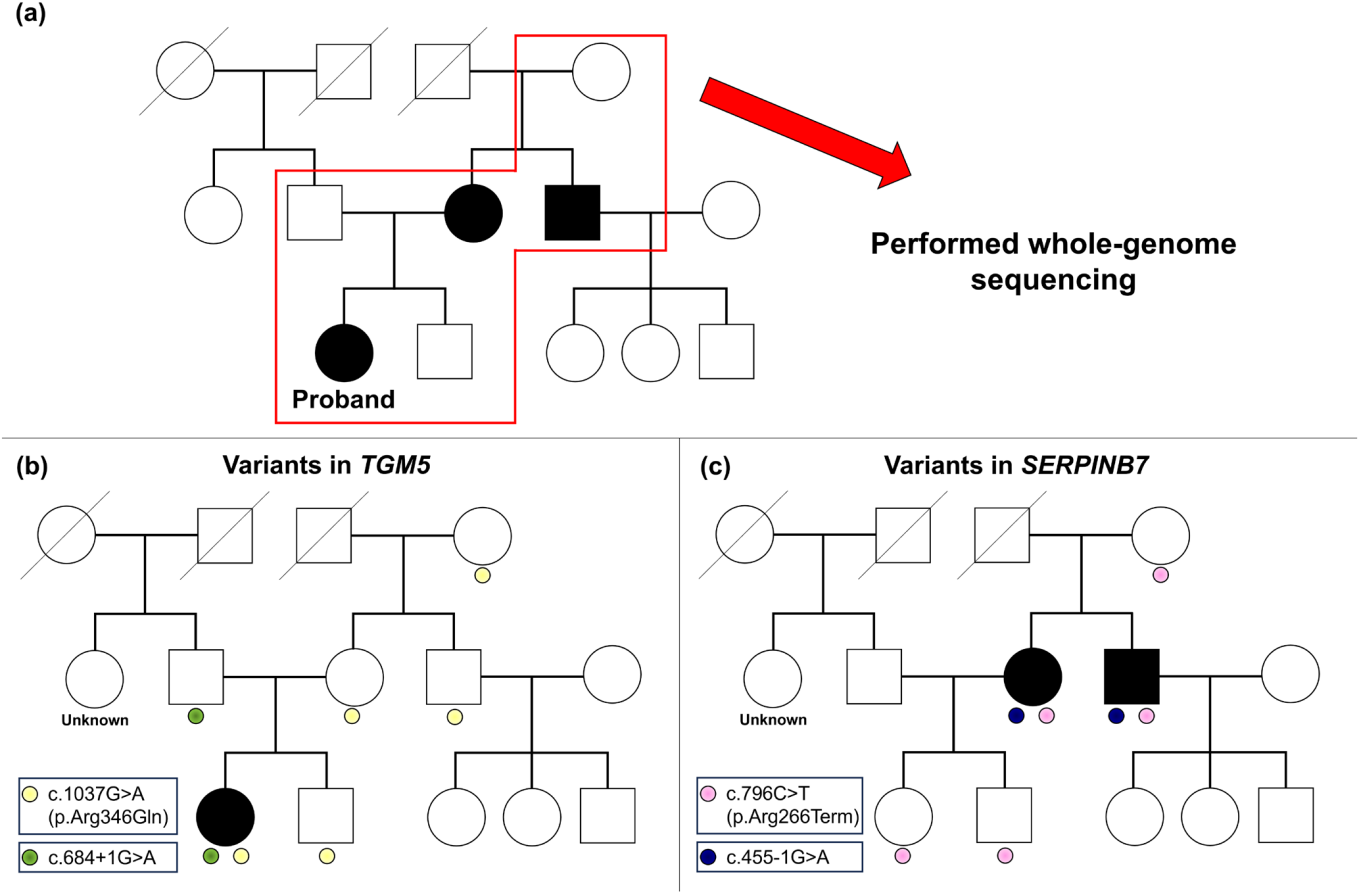
Pedigree of the patient's family. (a) Pedigree based on clinical abnormalities in acral regions. WGS was performed on blood DNA samples from six individuals enclosed in a red frame. (b) Pedigree showing APSS based on suspected causative variants in *TGM5*. (c) Pedigree showing NPPK based on pathogenic variants in *SERPINB7*.

Her family history revealed that her mother and maternal uncle exhibited hyperkeratosis and scales on their palms and soles (Figure 2), with occasional episodes of hand and foot skin peeling, especially after exposure to high humidity, although these symptoms could not be replicated experimentally. Her father, brother, and maternal grandmother showed no skin peeling or other abnormalities, and her maternal grandfather was deceased. There was no reported consanguinity in her parents or grandparents. Given the acral skin symptoms in multiple maternal family members, autosomal‐dominant inheritance was also hypothesized (Figure 3a).

To elucidate the genetic background, we performed whole‐genome sequencing (WGS) on the patient's family members with approval from the institutional review board of Self‐Defense Forces Central Hospital (Setagaya, Tokyo, Japan; approval number: 04‐017) (see Appendix S1). Protocols followed the Declaration of Helsinki, with written informed consent obtained. The patient was found to have compound heterozygous variants c.1037G>A (p.Arg346Gln; rs769556674) and c.684 + 1G>A in *TGM5* (rs755087362), inherited from her mother and father, respectively (Figure 3b). Both variants were rare, with minor allele frequencies (MAFs) <0.01% in Japanese^6^ and other populations^7^ and had high CADD v1.7 scores^8^ (32 [c.1037G>A] and 34 [c.684 + 1G>A]), indicating a high likelihood of altering protein function. Thus, she was diagnosed as the first Japanese case of APSS.

Her mother and maternal uncle were found to have only one suspected causative variant (c.1037G>A) in *TGM5*. However, they had compound heterozygous pathogenic variants^4^ c.796C>T (p.Arg266Term; rs142859678) and c.455‐1G>A (rs577442939) in *SERPINB7* (Figure 3c), leading to a diagnosis of NPPK. The patient had a heterozygous c.455‐1 G>A variant, but, no other rare variants (MAF < 1%) were found in *SERPINB7*, including in the non‐exonic regions.

## 3 DISCUSSION

APSS was first described in English literature in the late 1990s.^2^ APSS is characterized by blister formation and superficial peeling of the skin of the hands and feet, leaving residual painless erythema and healing without scarring.^2^ Symptoms worsen due to heat, humidity, and physical factors such as trauma or friction.^2^ The histopathology of APSS shows mild hyperkeratosis and splitting of the epidermis between the stratum granulosum and corneum.^9^ Treatment is generally symptomatic and supportive, with topical emollients and keratolytic agents offering protection from heat, humidity, and trauma, particularly pressure and friction.^10^

Although APSS is a clinically and genetically heterogeneous syndrome and given that its genetic background has not been fully revealed,^9^ there have been reports that homozygous or compound‐heterozygous dysfunctional variants of either *TGM5* ^9^, ^11^ or *CSTA* ^12^, ^13^ can cause APSS. *TGM5* encodes transglutaminase 5 (TG5), which has a protein cross‐linking function and is strongly expressed in epidermal granular cells.^14^ TG5 plays a pivotal role in forming the cornified cell envelope and maintaining cell–cell adhesion between the stratum corneum and stratum granulosum,^9^, ^15^ therefore the abolition of TG5 activity in the skin leads to skin peeling. The variant c.337G>T (p.Gly113Cys; rs112292549) in *TGM5* is a founder mutation^11^ with MAF of 0.38% in European population.^7^ On the contrary, c.337G>T in *TGM5* was not found in 19 936 East Asian individuals,^7^ which explains the rarity of APSS in East Asia. The two genetic variants in *TGM5* identified as suspected causative variants in this APSS case are extremely rare not only in Japanese^6^ but also in other populations.^7^ These data suggest that the phenotype of the case was likely a result of the simple coincidence of inheriting rare alleles. In other words, our findings do not support the existence of an endemic distribution of APSS in Japan. *CSTA* encodes cystatin A, which is a component of the cornified cell envelope.^12^ APSS secondary to *CSTA* variants shows diffuse fine cutaneous scaling, termed exfoliative ichthyosis, and acral peeling.^13^ To date, seven pedigrees with APSS due to homozygous variants in *CSTA* have been reported, with six of these cases associated with parental consanguinity.^13^

In contrast to APSS, NPPK is a relatively common keratoderma in Japanese and other East Asian populations due to founder mutations in *SERPINB7*, which encodes the serine protease inhibitor B7.^4^ NPPK is characterized by well‐demarcated diffuse hyperkeratosis with redness, extending onto the dorsal surfaces of the palms and feet, as well as the Achilles tendon area.^3^, ^4^ The MAFs of founder mutations in *SERPINB7* are 0.93% in Japanese^6^ and 0.77% in East Asian^7^ populations for c.796C>T, and 0.17% in Japanese^6^ and 0.094% in East Asian^7^ populations for c.455‐1G>A. This APSS case did not show hyperkeratosis or redness in the palmoplantar regions, which suggests that the heterozygous c.455‐1G>A variant in *SERPINB7* does not modify the phenotype by haploinsufficiency. NPPK patients may also exhibit acral peeling skin,^5^ although the underlying mechanism is not fully understood. Cohen‐Barak et al.^5^ reported mislocalization of DSG1 (desmoglein 1) and DSC1 (desmocollin 1) in the suprabasal layer of the skin in a patient with NPPK, along with reduced expression of DSG1 and DSC1, and decreased intercellular adhesion in SERPINB7‐silenced keratinocytes in vitro. They hypothesized that the target proteases of SERPINB7 include kallikrein isoforms, which degrade corneodesmosome proteins.^5^ In our pedigree, the patient's mother and maternal uncle, both exhibiting plantar scales, reported occasional acral peeling since their youth. However, we could not confirm this peeling experimentally. This might suggest that acral peeling in NPPK becomes less prominent with age or xerosis.

Identifying candidate causative genetic variants that explain skin disorders in all family members is not straightforward when multiple models of genetic inheritance are hypothesized, as in the present case, therefore comprehensive genetic analyses, such as whole‐exome sequencing and WGS, are valuable in such complex situations.

In conclusion, we report the first sporadic Japanese case of APSS whose maternal family members are NPPK. Comprehensive genetic analysis is useful if it is possible that two different genodermatoses with a similar genotype exist in a pedigree.

## CONFLICT OF INTEREST STATEMENT

Toshihide Higashino received an award and complimentary support from Novogene for whole‐genome sequencing in this study. Akiharu Kubo is an Editorial Board member of Journal of Dermatology and a co‐author of this article. To minimize bias, he was excluded from all editorial decision‐making related to the acceptance of this article for publication. Other authors declare no conflict of interest.

## Supporting information


Appendix S1.
