# Supplementary material for: Coincidence of acral peeling skin syndrome and Nagashima‐type palmoplantar keratosis in a Japanese pedigree with acral skin peeling
Source: J Dermatol. 2024 Aug 12;52(3):505–9. doi: 10.1111/1346-8138.17422 (PMC11883852; doi:10.1111/1346-8138.17422)
Supplement: Supplementary file 1 — Appendix S1. [file JDE-52-505-s001.docx]

**Supplementary Methods for Whole-Genome Sequencing**

**1. Experimental Procedure**

**1.1. Sample Quality Control**

The genomic DNA was extracted from whole blood using QIAcube (QIAGEN N.V.). The quality, integrity, and purity of the DNA were evaluated using Qubit (Thermo Fisher Scientific Inc.) and agarose gel electrophoresis.

**1.2. Library Preparation and Sequencing**

The genomic DNA was randomly sheared into short fragments. These fragments were end-repaired, A-tailed, and ligated with Illumina adapters (Illumina Inc.). The adapter-ligated fragments were then PCR-amplified, size-selected, and purified.

The library was checked using Qubit, real-time PCR for quantification, and 2100 Bioanalyzer (Agilent Technologies, Inc.) for size distribution detection. Quantified libraries were pooled and sequenced on NovaSeq 6000 (Illumina Inc.), according to the effective library concentration and the required data amount.

**2. Bioinformatics Analysis**

**2.1. Raw Data**

The original fluorescence image files obtained from NovaSeq 6000 were transformed into short reads (raw data) by base-calling, recorded in FASTQ format, which includes sequences and corresponding sequencing quality information.

**2.2. Data Quality Control**

Sequence artifacts, such as reads containing adapter contamination, low-quality nucleotides, and unrecognizable nucleotides (N), pose barriers to reliable bioinformatics analysis. To mitigate these obstacles and ensure meaningful downstream analysis, we performed quality control with the following data processing steps:

(1) Discard a pair of reads if either read contains adapter contamination (>10 nucleotides aligned to the adapter, allowing ≤ 10% mismatches).

(2) Discard a pair of reads if more than 10% of bases are uncertain (read as N) in either read.

(3) Discard a pair of reads if the proportion of low-quality (Phred quality <5) bases is over 50% in either read.

Total read number, raw data, error rate, and percentage of reads with Q30 (bases with Phred-scaled quality scores >30) were calculated and summarized. Filtered reads were used as clean data for subsequent analysis.

**2.3. Sequence Alignment**

Clean data were mapped to the reference genome (b37/hg19/hg38) using Burrows-Wheeler Aligner (BWA) software^1^ to generate BAM files. Subsequently, Sambamba^2^ was used to sort BAM files according to chromosome position. Picard tools (https://broadinstitute.github.io/picard/) were utilized to merge BAM files and mark duplicate reads.

**2.4. Variant Detection**

GATK^3^ HaplotypeCaller was used to call single nucleotide variants (SNVs) and insertions and deletions (InDels). The GATK VariantFiltration module was used to filter SNVs and InDels with the following parameters:

**SNVs:** QualByDepth (QD) < 2.0, FisherStrand (FS) > 60.0,

RMSMappingQuality (MQ) < 40.0, HaplotypeScore > 13.0,

MappingQualityRankSum < -12.5, and ReadPosRankSum < -8.0

**InDels:** QD < 2.0, FS > 200.0, and ReadPosRankSum < -20.0

**2.5. Annotation and Identification of Variants**

ANNOVAR^4^ was used for variant annotation, including protein-coding changes, genomic regions affected by variants, allele frequency, and deleterious predictions. Databases used for this study were:

**Genes and regions annotation:** RefSeq^5^ and Gencode.^6^

**Variant filtering:** Variants with MAF > 1% were filtered from candidate variants using 1000 Genomes^7^, Exome Aggregation Consortium (ExAC),^8^ and Genome Aggregation Database (gnomAD)^9^.

**Deleterious predictions:** SIFT,^10^ PolyPhen^11^ and CADD.^12^

**2.6. Evaluation of Candidate Variants in This Study**

After selecting candidates for causative variants, the parameters related to variant calling by GATK^3^ were reevaluated to determine if Sanger sequencing was required for genotype validation. In this study, all candidate variants met the following criteria^13^ across all called samples, thus negating the need for Sanger sequencing:

FILTER=PASS, QUAL≥100, depth coverage≥20X, and variant fraction≥20%

**References**

1. Li H, Durbin R. Fast and accurate short read alignment with Burrows-Wheeler transform. *Bioinformatics*. 2009;**25**(14):1754-60.

2. Tarasov A, Vilella AJ, Cuppen E, Nijman IJ, Prins P. Sambamba: fast processing of NGS alignment formats. *Bioinformatics*. 2015;**31**(12):2032-4.

3. DePristo MA, Banks E, Poplin R, Garimella KV, Maguire JR, Hartl C, et al. A framework for variation discovery and genotyping using next-generation DNA sequencing data. *Nat Genet*. 2011;**43**(5):491-8.

4. Wang K, Li M, Hakonarson H. ANNOVAR: functional annotation of genetic variants from high-throughput sequencing data. *Nucleic Acids Res*. 2010;**38**(16):e164.

5. O'Leary NA, Wright MW, Brister JR, Ciufo S, Haddad D, McVeigh R, et al. Reference sequence (RefSeq) database at NCBI: current status, taxonomic expansion, and functional annotation. *Nucleic Acids Res*. 2016;**44**(D1):D733-45.

6. Frankish A, Diekhans M, Jungreis I, Lagarde J, Loveland JE, Mudge JM, et al. Gencode 2021. *Nucleic Acids Res*. 2021;**49**(D1):D916-D23.

7. Genomes Project C, Abecasis GR, Auton A, Brooks LD, DePristo MA, Durbin RM, et al. An integrated map of genetic variation from 1,092 human genomes. *Nature*. 2012;**491**(7422):56-65.

8. Lek M, Karczewski KJ, Minikel EV, Samocha KE, Banks E, Fennell T, et al. Analysis of protein-coding genetic variation in 60,706 humans. *Nature*. 2016;**536**(7616):285-91.

9. Karczewski KJ, Francioli LC, Tiao G, Cummings BB, Alfoldi J, Wang Q, et al. The mutational constraint spectrum quantified from variation in 141,456 humans. *Nature*. 2020;**581**(7809):434-43.

10. Ng PC, Henikoff S. SIFT: Predicting amino acid changes that affect protein function. *Nucleic Acids Res*. 2003;**31**(13):3812-4.

11. Adzhubei IA, Schmidt S, Peshkin L, Ramensky VE, Gerasimova A, Bork P, et al. A method and server for predicting damaging missense mutations. *Nat Methods*. 2010;**7**(4):248-9.

12. Rentzsch P, Witten D, Cooper GM, Shendure J, Kircher M. CADD: predicting the deleteriousness of variants throughout the human genome. *Nucleic Acids Res*. 2019;**47**(D1):D886-D94.

13. Arteche-Lopez A, Avila-Fernandez A, Romero R, Riveiro-Alvarez R, Lopez-Martinez MA, Gimenez-Pardo A, et al. Sanger sequencing is no longer always necessary based on a single-center validation of 1109 NGS variants in 825 clinical exomes. *Sci Rep*. 2021;**11**(1):5697.
